# MEALTIME EXPERIENCES DURING AML/MDS TREATMENT: LONGITUDINAL QUALITATIVE STUDY PRELIMINARY RESULTS

**DOI:** 10.1093/geroni/igae098.4267

**Published:** 2024-12-31

**Authors:** Victoria Crowder, Youngmin Cho, Chiao-Hsin Teng, Melissa Batchelor, Anna Beeber, Lorinda Coombs, Daniel Richardson, Ashley Bryant

**Affiliations:** University of North Carolina at Chapel Hill, Chapel Hill, North Carolina, United States; Johns Hopkins University, Baltimore, Maryland, United States; National Health Research Institutes, Miaoli, Miaoli, Taiwan (Republic of China); George Washington University, Washington, District of Columbia, United States; Johns Hopkins University, Baltimore, Maryland, United States; University of North Carolina at Chapel Hill, Chapel Hill, North Carolina, United States; University of North Carolina at Chapel Hill, Chapel Hill, North Carolina, United States; University of North Carolina at Chapel Hill, Chapel Hill, North Carolina, United States

## Abstract

Older adults (aged ≥60) with acute myeloid leukemia (AML) and myelodysplastic syndromes (MDS) face varied experiences related to eating and mealtimes during treatment with hypomethylating agents and Venetoclax (HMA+VEN). Family/friend carepartners may provide extensive support to patients during this time and undergo challenges of their own. A gap exists in our understanding of eating and mealtime experiences over time, and how this affects quality of life (QOL); further information is crucial to guide patients, clinicians and carepartners in their management. We aimed to understand patient’s eating and mealtime experiences with AML/MDS and their carepartners during Cycles 1 and 2 of HMA+VEN treatment, the efforts made to address challenges, and the impacts on QOL. We conducted semi-structured interviews with 11 older adult patients and ten carepartners at Cycles 1 and 2 and conducted directed content analysis with the Adaptive Leadership Framework for Chronic Illness. We identified five preliminary categories: (1) adaptive challenges for patients in eating and mealtimes, (2) changes with eating and mealtimes over Cycles 1 and 2, (3) adaptive challenges for carepartners in care of patients, (4) how they collaboratively address challenges, and (5) the impact of experiences or changes in eating and mealtimes on QOL. Findings highlight and expand our knowledge of eating/mealtime experiences during chemotherapy for patients with AML/MDS and carepartners, including their experiences with appetite loss, adaptations of meals, and the extensive involvement of carepartners. These findings may guide future intervention development to support symptom management, enhance support and services, and maintain QOL.

